# Consensus Report of Group 4 of the 1st Global Consensus for Clinical Guidelines for the Rehabilitation of the Edentulous Maxilla: Conventional Dentures, Implant Overdentures and Implant‐Supported Fixed Dental Prostheses

**DOI:** 10.1111/clr.70069

**Published:** 2026-02-24

**Authors:** Charlotte Stilwell, Ronald E. Jung, Florian Beuer, Giulia Brunello, Jae‐kook Cha, Jeanette Chua Keng Ling, Donald A. Curtis, Karim El‐Kholy, Brian Goodacre, Lisa Heitz‐Mayfield, Mohit Kheur, Sunjai Kim, Hisatomo Kondo, Purnima Kumar, Alejandro Lanis, David Norre, Bjarni Pjetursson, Srinivasa Rao Bhasmey, Irena Sailer, Misi Si, Amerian Sones, Franz J. Strauss, Stefan Wolfart, Nicola U. Zitzmann

**Affiliations:** ^1^ Division of Gerodontology and Removable Prosthodontics, University Clinics of Dental Medicine University of Geneva Geneva Switzerland; ^2^ Center for Dental Medicine, Clinic of Reconstructive Dentistry University of Zürich Zürich Switzerland; ^3^ Department of Prosthodontics, TMJ and Geriatric Dentistry Charité University of Medicine Berlin Germany; ^4^ Department of Oral Surgery University Hospital of Düsseldorf Düsseldorf Germany; ^5^ Department of Orthodontics and Dentofacial Orthopaedics Charité ‐ Universitätsmedizin Berlin, Corporate Member of Freie Universität Berlin and Humboldt‐Universität zu Berlin Berlin Germany; ^6^ Department of Periodontology, Research Institute for Periodontal Regeneration Yonsei University College of Dentistry Seoul South Korea; ^7^ Ancora Imparo, Private Education Center Kuala Lumpur Malaysia; ^8^ Faculty of Dentistry, Department of Periodontology University of Malaya Kuala Lumpur Malaysia; ^9^ Department of Preventive and Restorative Dental Sciences, School of Dentistry University of California, San Francisco San Francisco California USA; ^10^ Department of Periodontology Columbia University College of Dental Medicine New York New York USA; ^11^ Department of General Dentistry, School of Dentistry Loma Linda University Loma Linda California USA; ^12^ Private Practice Upland California USA; ^13^ International Research Collaborative, Oral Health and Equity, School of Human Anatomy and Biology The University of Western Australia Crawley Western Australia Australia; ^14^ Faculty of Medicine and Health, School of Dentistry The University of Sydney Sydney New South Wales Australia; ^15^ Department of Prosthodontics and Implantology M A Rangoonwala Dental College and Research Centre Pune India; ^16^ Department of Prosthodontics, Gangnam Severance Dental Hospital, College of Dentistry Yonsei University Seoul Republic of Korea; ^17^ Department of Fixed Prosthodontics and Oral Implantology, School of Dentistry Aichi Gakuin University Nagoya Japan; ^18^ Department of Periodontics and Oral Medicine University of Michigan School of Dentistry Ann Arbor Michigan USA; ^19^ Department of Oral and Maxillofacial Implantology University of Chile School of Dentistry Santiago Chile; ^20^ Private Practice Overijse Belgium; ^21^ Faculty of Odontology, Department of Reconstructive Dentistry University of Iceland Reykjavik Iceland; ^22^ Sri Siddhartha Dental College Tumkur India; ^23^ Department of Oro‐Facial Rehabilitation, Division of Fixed Prosthodontics and Biomaterials, Clinic of Dental Medicine University of Geneva Geneva Switzerland; ^24^ Key Laboratory of Oral Biomedical Research of Zhejiang Province Stomatology Hospital, School of Stomatology, Zhejiang University School of Medicine, Zhejiang Provincial Clinical Research Center for Oral Diseases Hangzhou China; ^25^ Department of Comprehensive Dentistry Texas A&M University, College of Dentistry College Station Texas USA; ^26^ Clinic of Reconstructive Dentistry, Center of Dental Medicine University of Zurich Zurich Switzerland; ^27^ Universidad Autonoma de Chile Santiago Chile; ^28^ Department of Prosthodontics and Biomaterials RWTH Aachen University Aachen Germany; ^29^ Department of Reconstructive Dentistry, University Center for Dental Medicine Basel (UZB) University of Basel Basel Switzerland

**Keywords:** complete denture, dental implants, edentulous maxilla, implant overdentures, implant‐supported fixed prostheses dentures

## Abstract

**Objectives:**

The 1st Global Consensus for Clinical Guidelines (GCCG) in Implant Dentistry introduced an innovative, evidence‐based, patient‐centred approach to developing practical recommendations for the rehabilitation of the edentulous maxilla. Within this framework, Group 4 focused on formulating clinical recommendations for the planning and delivery of conventional dentures (CD), implant overdentures (IOD) and implant‐supported fixed complete dentures (IFCD) for patients with an edentulous maxilla.

**Materials and Methods:**

Group 4 followed the S2k‐level guideline framework of the Association of the Scientific Medical Societies in Germany (AWMF), applying a structured nominal group technique. The evidence base included two systematic reviews synthesising patient‐reported outcomes (PROs) and clinician‐reported outcomes (ClinROs) and their respective measures in patients with an edentulous maxilla. In addition, single‐round international surveys were conducted involving expert clinicians, patients and cross‐disciplinary experts. Draft recommendations were developed during the in‐person consensus meeting in Boston (June 16–18, 2025) and finalised through anonymous plenary voting. Consensus thresholds were predefined at ≥ 75% and ≤ 95% agreement for a consensus and > 95% agreement for a strong consensus.

**Results:**

Group 4 participants formulated 14 clinical recommendations covering five stages of treatment: (i) patient selection, (ii) diagnostics, (iii) treatment planning, (iv) treatment procedures and (v) maintenance. During plenary voting, 6 recommendations reached consensus and eight achieved strong consensus. Voting participation per recommendation ranged from 60 to 84 participants (mean 75).

**Conclusions:**

Group 4 consensus statements provide structured guidance on the use of removable and fixed implant‐supported dental prostheses for the rehabilitation of the edentulous maxilla. The recommendations reflect current evidence, expert opinion on clinical practice across disciplines and patient perspectives. Key evidence gaps—especially regarding standardised PROMs, long‐term outcomes and maintenance protocols—identify priorities for future clinical research.

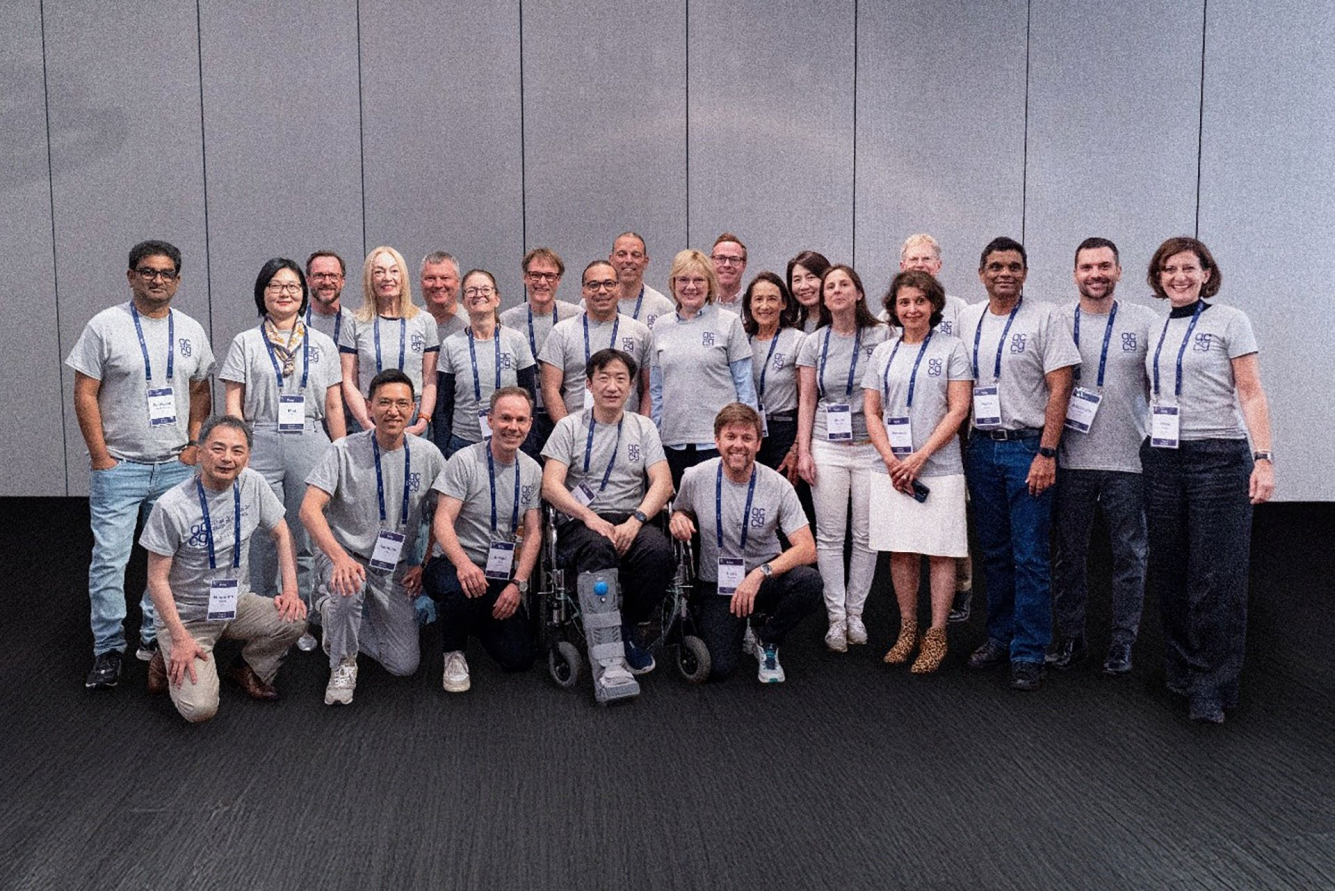

## 1 Introduction

The 1st Global Consensus for Clinical Guidelines (GCCG) in Implant Dentistry introduced an innovative, evidence‐based approach to consensus‐building in implant dentistry. The initiative focused on the rehabilitation of the edentulous maxilla, with the goal of developing patient‐centred and practical clinical recommendations. Working Group 4 focused on the prosthodontic rehabilitation of the edentulous maxilla.

## 2 Preamble

Three main options exist for the rehabilitation of the edentulous maxilla: a conventional denture (CD), an implant overdenture (IOD), either implant‐retained or implant‐supported, and an implant‐supported fixed dental prosthesis (IFDP), also termed implant‐supported complete denture (IFCD) (Jawad 2024; Sadowsky and Zitzmann 2016; Layton et al. 2023).

CDs rely fully on mucosal support, stability from residual ridge form, retention from optimal denture‐base fit and peripheral seal (suction) (Müller 2014). While maxillary CDs generally perform better than mandibular CDs, their function may be negatively affected by a reduction in patient‐related psychomotor ability or hyposalivation. Progressive ridge resorption, reduced denture‐bearing area, maxillary–mandibular discrepancies and unfavourable occlusal loading can also impact CD function (Celebic et al. 2003; McCord and Grant 2000; Lin, Chen, et al. 2026). Compared to implant‐supported alternatives, CDs are less expensive, less invasive (no surgery needed) and require shorter treatment time.

IODs typically rely on both mucosal and implant support. Mucosal support varies depending on the number and distribution of implants (Zitzmann and Marinello 1999a). Stability is influenced by the residual ridge form, while retention derives from denture‐base extension and fit and implant anchorage. IODs in the edentulous maxilla can be implant‐retained (e.g., unsplinted stud attachments) or implant‐supported (e.g., bar‐splint), while mucosal support varies depending on the number and distribution of implants (Zitzmann and Marinello 1999a). Stability is further influenced by the residual ridge form, while retention derives from denture‐base extension and fit and implant anchorage. The literature supports the use of four or more implants, either splinted by bar or unsplinted with stud attachments or telescopic crowns (Brandt et al. 2019; Messias et al. 2021; Zou et al. 2013). When using stud abutments, the ability to compensate for divergent implant axes is limited by the specific retention element of the system, typically to about 15°–25°. In contrast, telescopic crowns and bar splints allow for the compensation of divergent implant angulations, while providing a distinct path of insertion that facilitates prosthesis insertion and retrieval by the patient without assistance. An IOD bar can also be designed with distal cantilevers when implant position is restricted to the anterior region, particularly in Class II jaw relations (Zitzmann et al. 2016).

Compared to a maxillary CD, IODs are more expensive, invasive, need careful material selection when dealing with limited restorative space and require more maintenance. IODs offer the advantage over CDs of reduction of palatal coverage in response to a patient wish and subject to the anatomical situation (Heydecke et al. 2003). Compared to an IFCD, an IOD is less costly, requires fewer implants, involves less complex surgery, is easier to clean and offers more options for prosthetic design (Sailer et al. 2022). These include the advantage of individual design of the denture flanges for restoring facial and lip support as well as for satisfying phonetic requirements, which vary depending on alveolar ridge resorption (Zitzmann and Marinello 1999a, 1999b; Heydecke et al. 2003).

IFCDs rely exclusively on implants for support and retention. As a fixed dental prosthesis that is not removable by the patient, IFCDs must be designed without prostheses flanges that could impede access for daily cleaning as well as professional monitoring and maintenance. By implication, this also means that a buccal flange to support the lips and facial profile is not feasible and that no palatal extension can be used to optimise phonation in edentulous jaws with advanced resorption. IFCDs involve higher financial cost, greater surgical complexity, longer treatment times and more demanding hygiene protocols.

Most studies on the edentulous maxilla focus primarily on the optimal number of implants required for maxillary rehabilitation (Di Francesco et al. 2019; Daudt Polido et al. 2018; Messias et al. 2021; Slot et al. 2023). Furthermore, there are few studies documenting IOD rehabilitation of the edentulous maxilla (Schimmel et al. 2023), while advantages including improved oral function and oral health‐related quality of life for IODs and IFCDs compared to CDs in the mandible are well documented (Hartmann et al. 2020; Yao et al. 2018).

With CDs remaining a prosthetic reality for many patients worldwide, it is interesting to note that one study reported no statistically significant difference in patient satisfaction between a maxillary CD and an IOD (Thomason et al. 2007). A recent prospective, observational study even found that some patients preferred to continue with their new maxillary CD rather than proceed to IOD treatment (Zhang et al. 2019). Still, a recent consensus statement concluded that the use of dental implants to retain/support dental prostheses in the mandible and/or the maxilla improves overall patient‐reported outcomes (PROs) in edentulous patients wearing CDs (Schimmel et al. 2023; Srinivasan et al. 2023). Recent evidence shows IODs in the maxilla significantly enhance patient satisfaction and oral health‐related quality of life compared to CDs (Lin, Chen, et al. 2026).

Studies evaluating PROs following restoration of the edentulous maxilla with either IOD or IFCD (i.e., chewing function, phonetics, aesthetics and overall satisfaction) are equivocal (Yao et al. 2018). It is therefore important that the treatment option selected should be based on multiple factors using a patient‐centred shared decision‐making process.

Given the limited and heterogeneous evidence and the potential challenges, complexities and risks involved in each patient case (Müller 2014; Emami et al. 2014; Gallucci et al. 2016; Dawson et al. 2021), this consensus sought to develop recommendations from a prosthetic perspective for the rehabilitation options CD, IOD and IFCD.

## 3 Methodological Framework and Consensus Procedures of the 1st GCCG

This Working Group was one of four formed within the 1st GCCG for the Rehabilitation of the Edentulous Maxilla, following an S2k‐level framework of the Association of the Scientific Medical Societies in Germany (AWMF). Group 4 included 24 of the 105 participating experts. These 24 experts represented three specialties: prosthodontics (15), periodontics (7) and oral surgery/oral maxillofacial surgery (2).

Group recommendations were developed through a structured, multi‐phase process combining systematic evidence synthesis with input from expert clinicians, patients and cross‐disciplinary experts. Preparatory work included systematic reviews on PROs, clinician‐reported outcomes (ClinROs) their respective measures (Lin, Chen, et al. 2026; Park et al. 2026), as well as structured surveys of clinicians, patients and other cross‐disciplinary experts (Schoenbaum et al. 2026; Strauss et al. 2026; Brunello, Strauss, et al. 2026; Lin, Brunello, et al. 2026; Lin, Strauss, et al. 2026). These data provided the foundation for Working Group discussions during the in‐person meeting in Boston (June 16–18, 2025).

The PROs and ClinROs and their respective measures identified through the systematic reviews and the expert clinician, patient and cross‐disciplinary surveys informed the first round of a three‐round Delphi process (Khodyakov et al. 2023) to define a Core Outcome Set (COS) for edentulous maxilla rehabilitation (Brunello, Lin, et al. 2026). This set was refined over two additional rounds during the consensus conference and subsequently adopted for the formulation of the final recommendations. During the Delphi process, no direct discussion between experts took place; consensus was based on response distribution across survey rounds in an iterative process.

Using the Nominal Group Technique (Harvey and Holmes 2012), each recommendation was developed and refined through individual review, prioritisation voting and moderated discussion. Recommendations were primarily derived from the results of the structured surveys and reviewed in the context of the available evidence and categorised as

- Aligned with current evidence (survey results consistent with available data)
- Not aligned with current evidence (survey results diverged from available data) or
- Could not be evaluated due to a lack of existing evidence.

Final consensus was reached via anonymous plenary voting, with predefined thresholds of agreement: strong consensus (> 95%); consensus (≥ 75% and ≤ 95% %); no consensus (< 75%). Conflicts of interest (CoI) were disclosed as per ICMJE standards and abstentions were documented. The full methodological framework is detailed in the umbrella paper (Schwarz et al. 2026).

## 4 Summary of the Reported Clinician‐ and Patient‐Reported Outcomes

In preparation for the 1st GCCG in Implant Dentistry, eight systematic reviews were conducted to identify and evaluate available evidence on PROs, ClinROs and their respective measures. Two of these systematic reviews focused on findings from studies evaluating CDs, IODs and IFCDs (Park et al. 2026; Lin, Chen, et al. 2026). These PROs and ClinROs and related measures were incorporated in the discussions of Working Group 4 and are summarised below.

### 4.1 PROs

PROs addressed overall satisfaction with prostheses, changes in satisfaction over time, adaptation to new prostheses type (Park et al. 2026) and functional outcomes. Specific domains included stability, comfort, ease of cleaning, speech clarity and aesthetic appearance (Lin, Chen, et al. 2026) as well as denture complaints and chewing ability (Park et al. 2026).

Across multiple studies, patients reported higher oral health‐related quality of life (OHRQoL) and satisfaction with IODs compared to CDs, largely due to fewer functional complaints (e.g., looseness, pain) and improved stability and function (Lin, Chen, et al. 2026).

### 4.2 Patient‐Reported Outcome Measures (PROMs) for the Assessment of PROs

Many studies used validated tools such as short‐form OHIP surveys (OHIP‐14 and OHIP‐20) or the McGill Denture Satisfaction questionnaire as PROMs to assess PROs (Lin, Chen, et al. 2026; Park et al. 2026).

Other PROMs included visual analogue scales (VAS) assessing domains such as comfort, chewing ability and speech (Lin, Chen, et al. 2026; Park et al. 2026) as well as global satisfaction questionnaires evaluating aesthetics, prosthetic stability and ease of cleaning (Lin, Chen, et al. 2026; Park et al. 2026).

Follow‐up assessments ranged from baseline (with CD) to 3–12 months after conversion to IOD (Lin, Chen, et al. 2026).

### 4.3 Clinician‐Reported Outcome Measures (CROMs) for the Assessment of ClinROs

CROMs were primarily limited to objective measures, covering implant and prosthesis survival and success, maintenance requirements and biological parameters (e.g., peri‐implant bone loss, peri‐implantitis, peri‐implant mucositis) (Lin, Chen, et al. 2026; Park et al. 2026).

Park et al. (2026) reported that in studies involving IODs, both implants and prosthesis survival rates ranged from 80% to 100%. Success criteria, however, varied widely across studies with more than five years' follow‐up, with prosthesis success rates (55.0%–90.0%) typically lower than implant success rates (78.2%–95.0%).

Maintenance requirements frequently included activation or replacement of the matrix of the attachment system, relining, of the prosthesis and management of prosthetic complications including fractures of the denture base. Biological parameters were consistently assessed, with peri‐implant soft tissue health status and crestal bone levels reported across studies (Lin, Chen, et al. 2026; Park et al. 2026). Follow‐up intervals varied considerably, ranging from baseline to 1, 2, or 5 years, with annual evaluations being most common (Park et al. 2026).

Only a few studies reported subjective CROMs such as clinician rating of overall prosthetic fit or complexity and none of the studies included subjective CROMs specifically in the context of maxillary IOD therapy (Park et al. 2026).

## 5 Summary of the Key Findings from the Surveys

Group 4 analysed results from three single‐round surveys: one involving 121 expert clinicians (of 230 invited from 41 countries, response rate 52%) on implant‐supported fixed and removable dental prostheses (Lin, Chen, et al. 2026) and two surveys involving 41 patients (of 68 invited from 29 countries, response rate 60%) and 21 cross‐disciplinary experts (of 68 invited from 27 countries, response rate 31%), respectively, on the rehabilitation of the edentulous maxilla (Lin, Brunello, et al. 2026). The findings were incorporated into the discussions of Working Group 4 and are presented below according to the major clinical stages defined by the GCCG framework: patient selection, diagnostics, treatment planning, treatment procedures and maintenance.

### 5.1 Patient Selection

When asked under what circumstances they would consider IODs retained by stud attachments or bar splints, most experts (64.5%) reported using them to compensate for insufficient alveolar volume lip support. A third of the experts (33.1%) cited patient finances as the main reason. A smaller proportion indicated IODs as their preferred option whenever possible (16.5%), whereas 15.7% reported they would never prefer this option.

Both patients (82.9%—consensus) and cross‐disciplinary experts (76.2%—consensus) showed a clear preference for fixed over removable prostheses. Patients prioritised aesthetics (95.1%—strong consensus), comfort (73.2%) and function (70.7%) as primary concerns. There was consensus amongst the cross‐disciplinary experts for chewing (90.5%), aesthetics (85.7%) and cleaning ability (81%) as the primary concerns.

### 5.2 Diagnostics

For tooth positioning, consensus amongst the experts supported the facial midline and upper lip (94.2%) as the most reliable reference points. Secondary landmarks such as the upper and lower lips (67.8%) and eyes/bi‐pupillary line (66.9%) did not reach consensus. There was consensus amongst patients and the cross‐disciplinary experts favoured the use of CBCT (92.7% and 90.5%, respectively) for planning implant therapy.

### 5.3 Treatment Planning

In line with the preference for fixed dental prostheses, there was consensus from both the patients (80.5%) and the cross‐disciplinary experts (85.7%) for being open to undergoing hard tissue augmentation procedures and to accept the additional cost involved.

Regarding implant design, most experts preferred bone‐level implants (66.1%) and agreed that 3.5 mm should be the minimum diameter in a normal occlusal scenario (81%—consensus). The first molar position was identified as the preferred most distal implant site for a maxillary IFCD in cases with sufficient bone (81.8%—consensus).

For IFCD design, there was consensus from the experts in favour of screw‐retained prostheses (93.4%). Other design features, such as angled screw channel systems (59.5% “sometimes”), multi‐abutments (62% “always”), one‐piece prostheses rather than segmented prostheses (54.4% “mostly”) achieved only majority agreement. No consensus was reached on prosthetic material, although a majority supported considering a milled titanium framework sometimes (60.3%) and 66.1% reported always using titanium abutments or bases.

### 5.4 Treatment Procedures

Expert responses were divided on the timing of provisional prosthesis delivery (immediate vs. delayed) with no clear majority. Most experts (61.2%) reported only occasionally converting a conventional removable denture into a fixed provisional denture.

The preference of experts for analogue or digital impression techniques did not reach a consensus. Patients and cross‐disciplinary experts similarly did not express a strong preference for one impression approach over another. There was no agreement amongst the experts regarding the maximum number of implants to include when using an intraoral scanner for fabricating a master cast.

### 5.5 Maintenance

Experts most frequently recommended super floss (72.7%), interdental/proxy brushes (72.7%), electric toothbrushes (61.2%) and water flossers (58.7%) as hygiene aids. Patients (87.8%—consensus) and cross‐disciplinary experts (85.7%—consensus) favoured toothbrushes with patients also supporting water flossers (70.7%) and cross‐disciplinary experts supporting interdental brushes (71.4. %) and water flossers (58.1%).

Regarding removal of definitive prostheses for professional evaluation, hygiene, or screw replacement, expert responses were highly variable with no consensus on optimal intervals for removal (ranging from every visit through various year points to never). For retorquing prosthetic screws, most experts reported never or almost never (55.3%) doing so.

When asked about complications with IFCDs, experts most frequently reported fractures of veneering porcelain (58.7%). Other issues, including screw loosening (23.1%), debonding of titanium bases or abutments (9.1%), framework fractures (8.3%) and broken screws (0.8%) did not reach agreement. Most patients (65.9%) and cross‐disciplinary experts (66.7%) indicated they would use a protective occlusal guard daily.

Most patients expressed concern about implant failure (58.5%), while cross‐disciplinary experts were concerned about implant loss and adequate access for cleaning (71.4% for both). Nonetheless, there was consensus in both groups (92.7% and 90.5%) for expecting implants to last more than 10 years and that they should not fail within the first five years. Most respondents in both groups also felt well informed by their dentist regarding what to expect and the longevity of their implants and prostheses (73.2% of patients and 71.4% of cross‐disciplinary experts).

## 6 Group Recommendations

To enhance clinical applicability, 14 recommendations were structured according to a logical treatment workflow for the rehabilitation of the edentulous maxilla. This sequence follows the major clinical stages defined by the GCCG framework: (i) patient selection, (ii) diagnostics, (iii) treatment planning, (iv) treatment procedures and (v) maintenance care. Each recommendation reflects the outcome of structured consensus discussions and has been classified according to its alignment with the current body of evidence. Of the 14 recommendations, six reached consensus during plenary voting and eight achieved strong consensus.

### 6.1 Patient Selection

Recommendation No. 1 (Aligned with current evidence)

**Table:** 

Domain	Patient assessment
Recommendation	In patients with edentulous maxillae, we suggest that the selection of a conventional denture or most appropriate implant‐supported/retained rehabilitation be based on: Comprehensive, structured assessments Objective measures of risk/benefit/complexity and anticipated adherence to compliance Patient understanding of treatment risk and shared responsibility for achieving and maintaining long‐term health Shared decision‐making process
Expert survey	Not addressed
Patient/cross‐disciplinary expert surveys	Not addressed
Supporting/contradicting literature	Both patient and provider preferences determine the selection of final prosthesis and patient‐driven choices play an influential role in determining satisfaction and treatment outcomes (Brennan et al. 2010). Shared decision‐making is an ethical imperative for involving patients properly in decisions about their care and essential conditions for achieving this include evidence‐based information, adequate guidance on pros and cons of treatment options and supportive culture for patient engagement (Elwyn et al. 2010). SAC Classification for determination of case complexity and risks as part of comprehensive and structured patient assessment (Dawson et al. 2021). Patients expressed similar satisfaction after six months with their respective IODs and IFCDs but IODs users experienced greater pre‐ to post‐treatment improvements in aesthetics, taste and speech (Zitzmann and Marinello 2000). In a crossover trial comparing patient satisfaction with removable or fixed implant‐supported prostheses, nine patients chose to keep the removable prosthesis whereas four preferred to keep the fixed prosthesis. These findings support patient‐driven choices as an important determinant of treatment planning (Heydecke et al. 2003). Overall patient satisfaction ratings for maxillary implant prostheses were not significantly higher than for complete dentures (Thomason et al. 2007). Evidence of higher satisfaction with IODs than CD for the mandible may not hold true for patients with edentulous maxilla. Edentulous patients also often opt for a fixed or removable rehabilitation on implants for specific reasons (De Bruyn et al. 2015; Emami et al. 2014). In a prospective study evaluating PROMs and treatment outcomes of 103 patients, 80 chose to continue with their new CD after 3–4 months of wear, therefore not taking up the option of an implant‐retained next step (Zhang et al. 2019). With no standardisation of PROMs between studies for edentulous patients restored with implant‐supported removable and fixed prostheses there is heterogeneity in reporting and interpreting outcomes (Yao et al. 2018). Social and socio‐dental dimensions are important modulators of acceptance. In a patient‐centred approach, specific questionnaires for identifying sociodemographic and clinical OHRQoL modulators are suggested as effective tools for accurately assessing the chances of success of prospective IODs (Lo‐Sardo et al. 2025). PROMs are heterogenous across studies, preventing clinically meaningful inferences. Standardised, validated and customisable outcome measures need to be developed for reporting all such studies (Yao et al. 2018; Park et al. 2026)
Lack of literature	N/A
Evidence	Based on 3 systematic reviews (Park et al. 2026; Thomason et al. 2007; Yao et al. 2018), 4 comparative studies (Brennan et al. 2010; Heydecke et al. 2003; Zhang et al. 2019; Zitzmann and Marinello 2000), 1 prospective observational study (Lo‐Sardo et al. 2025), 1 narrative review (De Bruyn et al. 2015) and 1 conceptual article on shared decision‐making (Elwyn et al. 2010)
Recommended ClinROs	Clinician's treatment success History of patient adherence/compliance
Recommended PROs	Aesthetic satisfaction Chewing function/comfort/discomfort Ease of cleaning/oral hygiene efficacy Patient overall satisfaction with treatment Patient‐reported complaints Prosthesis retention/stability Quality of life (Oral Health‐Related Quality of Life, OHRQoL)
Strength of Consensus	Agree: 95% (Consensus) Agree: 69/Disagree: 2/Abstain:1/Abstain (CoI): 1

### 6.2 Diagnostics

Recommendation No. 2 (Aligned with current evidence)

**Table:** 

Domain	Diagnostics
Recommendations	In patients with edentulous maxillae, we suggest defining the final prosthetic goal with the help of a facially driven tooth arrangement (diagnostic set‐up), which may be achieved by using either: The existing denture if it is satisfactory A new conventional or digital diagnostic set‐up and fabrication of a trial denture, followed by a physical try‐in The static and dynamic evaluation of the tooth arrangement is recommended, and any required modifications of the lower arch should be taken into consideration. The aim of the above is to achieve predictable long‐term aesthetics, phonetics and function and reduce the risks of complications
Expert survey	Consensus (94.2%) for the use of the facial midline and upper lip as primary reference points
Patient/cross‐disciplinary expert surveys	Consensus that patients considered aesthetics (92.5%) one of the most important treatment outcomes. Consensus that cross‐disciplinary experts considered chewing function (90.5%), aesthetics (85.7%) and ease of cleaning (81.0%) the primary outcome concerns
Supporting/Contradicting literature	Planning optimal aesthetics is needed for both fixed and removable implant prosthesis to fulfil the patient's preference. Implants should be placed to comply with the selected prosthetic solution and compromised solutions should be avoided (Zitzmann and Marinello 1999a). A lip‐tooth‐ridge classification can assist clinicians in establishing final prostheses design and identifying aesthetic risks in implant rehabilitation of the edentulous maxilla (Pollini et al. 2017). The findings of an analysis of the smile framework (including the relationship between facial and dental midlines, key anatomical landmarks and comparison between facial and golden proportions) emphasised the importance of integrating standardised aesthetic principles to achieve predictable and satisfactory outcomes across macro‐, mini‐ and micro‐aesthetic levels (Bagheri et al. 2025). A study of six different racial groups found no significant differences between genders for any of the seven frontal and six profile extraoral parameters. There were differences between the racial groups for two out of seven frontal and five prolife parameters (Owens et al. 2002)
Lack of literature	N/A
Evidence	Based on 3 observational studies (Bagheri et al. 2025; Owens et al. 2002; Pollini et al. 2017) and 1 narrative review (Zitzmann and Marinello 1999a)
Recommended ClinROs	Prosthesis success Prostheses retention/stability
Recommended PROs (PROMs)	Aesthetic satisfaction Ease of cleaning/oral hygiene efficacy Functional comfort Patient overall satisfaction with treatment Speech/phonetics/pronunciation function Quality of life (Oral Health‐Related Quality of Life, OHRQoL)
Strength of Consensus	Agree: 96% (strong consensus) Agree: 78/Disagree: 2/Abstain: 1/Abstain (CoI): 0

Recommendation No. 3 (Aligned with current evidence)

**Table:** 

Domain	Diagnostics
Recommendation	In patients with edentulous maxillae, if an implant‐supported/retained rehabilitation is indicated and considered by the patient, we suggest that 3D imaging (CT/CBCT) should be made with a diagnostic set‐up including radio‐opaque markers for visualisation of the planned tooth arrangement in relation to the underlying anatomic structures. The aim of the above is to ensure prosthetically driven implant positioning, thereby enhancing treatment outcomes and long‐term stability
Expert survey	Consensus/strong consensus (83.8%–95%) expert support for use of CT/CBCT for planning.
Patient/cross‐disciplinary expert surveys	Consensus amongst patients (92.7%) and cross‐disciplinary experts (90.5%) for the use of CBCT for planning
Supporting/contradicting literature	The relationships should be assessed using computed tomography (Zitzmann and Marinello 1999a, 1999b) The American Academy of Periodontology (AAP) supports the use of CBCT in the surgical management of dental implants. Furthermore, when susceptible tissues are properly shielded and the field of view is limited to the area of interest the radiation risk is considered low. (Mandelaris et al. 2017) EAO Delphi study considering trends in implant dentistry for the year 2030, reached consensus (84%) that CBCT‐3D technologies will be the main tool for pre‐surgical implant placement diagnosis together with direct digital restorative workflows (Sanz et al. 2019). A Latin American Delphi study reached a similar consensus that guided surgery will be more common (Alarcón et al. 2021). Stressing the importance of minimising unnecessary radiation exposure while maintaining diagnostic accuracy (Bornstein et al. 2014). Current concepts for the use of cone beam computed tomography imaging, before and after implant placement, in daily clinical practice and research and discussion of guidelines for the selection of three‐dimensional imaging. (Bornstein et al. 2017). Low‐dose CBCT protocols do not compromise objective image quality during any stage of implant therapy (Kaaber et al. 2024)
Lack of literature	N/A
Evidence	Based on 1 systematic review (Kaaber et al. 2024), 2 narrative reviews (Zitzmann and Marinello 1999a; Bornstein et al. 2017) and 4 consensus reports (Alarcón et al. 2021; Bornstein et al. 2014; Mandelaris et al. 2017; Sanz et al. 2019)
Recommended ClinROs	Surgical/intraoperative complications
Recommended PROs	Complications during treatment Functional comfort Pain Patient‐reported complaints
Strength of Consensus	Agree: 95% (consensus) Agree: 72/Disagree: 3/Abstain: 1/Abstain (CoI): 0

### 6.3 Treatment Planning

Recommendation No. 4 (Aligned with current evidence)

**Table:** 

Domain	Treatment planning
Recommendation	In patients with edentulous maxillae, as long as the patient is satisfied with the retention, aesthetics and function of their maxillary complete denture with clinically healthy supporting tissues, no further treatment is needed apart from routine maintenance. The patient should be fully advised on potential long‐term impact of conventional denture wear and the alternative implant treatment options.
Expert survey results	Not addressed
Patient/cross‐disciplinary expert surveys	Not addressed
Supporting/contradicting literature	The significant evidence of higher satisfaction with IODs than CDs for the edentulous mandible may not hold true for the patient with edentulous maxilla (De Bruyn et al. 2015). Overall patient satisfaction ratings given to the maxillary implant prostheses were not significantly higher than for complete dentures (Thomason et al. 2007). In a 5‐year prospective study, 80 out of 103 patients chose to continue with their new CD after 3–4 months of wear, rather than taking up the option of an IOD as next step (Zhang et al. 2019).
Lack of literature	NA
Evidence	Based on 2 systematic reviews (De Bruyn et al. 2015; Thomason et al. 2007) and 1 prospective observational study (Zhang et al. 2019)
Recommended ClinROs	History of patient adherence/compliance Plaque index/oral hygiene Prostheses success
Recommended PROs	Aesthetic satisfaction Chewing function/comfort/discomfort Ease of cleaning/oral hygiene efficacy Patient‐reported complaints Prosthesis retention/stability Speech/phonetics/pronunciation function Quality of life (Oral Health‐Related Quality of Life, OHRQoL)
Strength of Consensus	Agree: 97% (strong consensus) Agree: 77/Disagree: 1/Abstain: 0/Abstain (CoI): 1

Recommendation No. 5 (Aligned with current evidence)

**Table:** 

Domain	Treatment planning
Recommendation	In patients with edentulous maxillae, when an implant‐ supported/retained prosthesis is being considered, clinical assessment (e.g., facial and lip support, restorative space, palatal coverage, arch relationship and residual ridge morphology) and financial considerations are recommended for assessing patient readiness and treatment options.
Expert survey results	94.2% of experts confirmed the use of the facial midline and upper lip as primary reference points. Most of the experts also considered both lips as secondary reference points (67.8%).
Patient/cross‐disciplinary expert surveys	Not addressed
Supporting/contradicting literature	This observational study on 87 subjects identified the midline of the oral commissure as the closest reliable landmark to the midline of the face in smile (Bidra et al. 2009). The findings of an analysis of the smile framework (including the relationship between facial and dental midlines, key anatomical landmarks and comparison between facial and golden proportions) emphasised the importance of integrating standardised aesthetic principles to achieve predictable and satisfactory outcomes across macro‐, mini‐ and micro‐aesthetic levels (Bagheri et al. 2025). A study of 454 full face photographs found that the maxillary anterior teeth and premolars show in an average smile, the incisal curve of the teeth is parallel to the inner curvature of the lower lip and the incisal curve of the maxillary anterior teeth is close to or slightly touching the lower lip (Tjan et al. 1984). A randomised cross‐sectional study of 300 dried skulls found that the vertical and horizontal changes in alveolar process were highly significant and followed a predictable pattern (Cawood and Howell 1988). A lip‐tooth‐ridge classification can assist clinicians in establishing final prostheses design and identifying aesthetic risks in implant rehabilitation of the edentulous maxilla (Pollini et al. 2017). A study of six different racial groups found no significant differences between genders for any of the seven frontal and six profile extraoral parameters. There were differences between the racial groups for two out of seven frontal and five prolife parameters (Owens et al. 2002)
Lack of literature	N/A
Evidence	Based on 5 observational studies (Bagheri et al. 2025; Bidra et al. 2009; Cawood and Howell 1988; Owens et al. 2002; Tjan et al. 1984) and 1 narrative review (Pollini et al. 2017)
Recommended ClinROs	History of patient adherence/compliance Implant primary stability Plaque index/oral hygiene Presence of keratinised mucosa Prosthesis retention/stability Width of keratinised mucosa
Recommended PROs	Aesthetic satisfaction Chewing function/comfort/discomfort Patient‐reported complaints Prosthesis retention/stability Speech/phonetics/pronunciation function Quality of life (Oral Health‐Related Quality of Life, OHRQoL)
Strength of Consensus	Agree: 96% (strong consensus) Agree: 81/Disagree: 2/Abstain: 1/Abstain (CoI): 0

### 6.4 Treatment Procedures

Recommendation No. 6 (Aligned with current evidence)

**Table:** 

Domain	Treatment procedure
Recommendation	In patients with edentulous maxillae, we suggest the use of a prosthetically driven surgical template based on the clinically verified tooth arrangement to ensure prosthetically oriented 3D implant positioning that enables appropriate prosthesis design from an aesthetic, biologic and technical perspective
Expert survey results	Not addressed
Patient/cross‐disciplinary expert surveys	Not addressed
Supporting/contradicting literature	A surgical guide that comes from a planning with a primary wax‐up of the prosthesis leads to the placement of a dental implant that can be functionally and aesthetically rehabilitated (Abad‐Coronel et al. 2024). Proper implementation of static computer‐aided implant surgery may increase the level of agreement between planned and definitive implant 3D positions in the aesthetic zone, thereby enhancing the aesthetic outcomes of implant rehabilitation (Markovic et al. 2024). The accuracy of implant placement using a surgical guide was significantly higher than that of free‐hand implantation. (Hama and Mahmood 2023). In evaluating the accuracy between virtual planning of computer‐guided surgery and actual outcomes of dental implants in edentulous alveolar ridges, the greatest inaccuracy in angular deviation was observed in the maxilla (Marlière et al. 2018). While guided surgery demonstrates implant survival rates comparable to conventional protocols, the clinical demands on the surgeon remain no less than those encountered during conventional implant placement (Hultin et al. 2012)
Lack of literature	N/A
Evidence	Based on 2 systematic reviews (Abad‐Coronel et al. 2024; Hultin et al. 2012) and 2 reviews (Marlière et al. 2018; Markovic et al. 2024), 1 in vitro study (Hama and Mahmood 2023)
Recommended ClinROs	Implant primary stability Postoperative complications Presence of keratinised mucosa Surgical/intraoperative complications Width of keratinised mucosa
Recommended PROs	Pain Patient‐reported complaints Speech/phonetics/pronunciation function
Strength of Consensus	Agree: 91% (consensus) Agree: 67/Disagree: 6/Abstain: 1/Abstain (CoI): 0

Recommendation No. 7 (Aligned with current evidence)

**Table:** 

Domain	Treatment procedure
Recommendation	In patients with edentulous maxillae, receiving a fixed implant‐supported dental prosthesis, we suggest using an interim fixed prosthesis to thoroughly evaluate aesthetics, function, phonetics, occlusion, cleanability and soft tissue stability prior to fabrication of the final prosthesis. This enables incorporation of modifications into the definitive prosthesis and ensures enhanced treatment outcome and long‐term stability
Expert survey results	Most of the expert responses were divided between always converting conventional removable dentures into fixed provisionals (9.9%) and doing so sometimes (61.2%). The remaining experts (28.9%) reported never doing so
Patient/cross‐disciplinary expert surveys	A fixed provisional prosthesis was preferred by 58.5% of the patients whereas the responses amongst the cross‐disciplinary experts were more heterogenous with no majority preference for either fixed or removable
Supporting/contradicting literature	A fixed provisional restoration, inserted for a six‐month diagnostic period to evaluate and optimise aesthetics, biology and function can then serve as the ideal guide for the final restoration (Zitzmann and Marinello 1999a). Provisional prostheses help sculpt the peri‐implant mucosa and improve aesthetic outcomes. Immediate provisionals have also been shown to positively influence papilla preservation and soft tissue stability. For further patient convenience, provisional prostheses can also serve as back‐up, temporary replacement to definitive prostheses (Wittneben et al. 2014). Predictable long‐term implant rehabilitation outcomes were demonstrated for the edentulous maxilla using fixed provisional prosthesis and an immediate loading protocol (Toljanic et al. 2016). Patients reported significantly higher satisfaction when provisionals were used, even in the short term. Immediate loading with temporary prostheses has also shown high survival rates and better patient‐reported outcomes (Gallucci et al. 2018).
Lack of literature	N/A
Evidence (narrative)	Based on 2 systematic reviews (Gallucci et al. 2018; Wittneben et al. 2014), 1 narrative review (Zitzmann and Marinello 1999a) and 1 clinical trial (Toljanic et al. 2016).
Recommended ClinROs	Implant primary stability Mechanical/technical complications Plaque index/oral hygiene Post operative complications Presence of keratinised mucosa Prosthodontic maintenance events/complications Width of keratinised mucosa
Recommended PROs	Aesthetic satisfaction Chewing function/comfort/discomfort Complications during treatment/maintenance Ease of cleaning/oral hygiene efficacy Pain Prosthesis retention/stability Speech/phonetics/pronunciation function
Strength of Consensus	Agree: 95% (consensus) Agree: 74/Disagree: 2/Abstain: 2/Abstain (CoI): 0

Recommendation No. 8 (Aligned with current evidence)

**Table:** 

Domain	Treatment procedure
Recommendation	In patients with edentulous maxillae receiving implant overdentures (IODs), we suggest metal reinforcement, preferably with an open palatal design, to optimise patient comfort, prosthesis stability and long‐term durability. Additionally, the selection of appropriate retention elements (e.g., stud attachments, milled bars, or telescopic crowns) is made depending on implant position and available interocclusal space.
Expert survey	When considering IODs (i.e., stud attachments or bar splints) for the edentulous maxilla, a majority (64.5%) indicated they used this option when alveolar volume was insufficient for lip support and 33.1% reported using this option when patient finances dictated.
Patient/cross‐disciplinary expert surveys	There was consensus amongst both the patients (82.9% (*n* = 34)) and the cross‐disciplinary experts (76.2% (*n* = 16)) for preferring a fixed maxillary full‐arch prosthesis over a removable overdenture.
Supporting/contradicting literature	In a crossover trial, 13 patients with maxillary implants compared fixed implant prostheses to removable long‐bar retained overdentures, each worn for two months. Removable overdentures received significantly higher ratings for general satisfaction (*p* = 0.003), speech (*p* = 0.036) and ease of cleaning (*p* = 0.004) compared with fixed prostheses. At the end of the study, nine patients preferred the removable option, suggesting that maxillary implant‐supported overdentures may provide superior function (Heydecke et al. 2003). A variety of retention systems are available to retain an implant overdenture. Clinical and treatment planning considerations are involved in selecting the most appropriate retention system for patients, including understanding of how these systems work, and their advantages and disadvantages (Laverty et al. 2017). VAS results at three years suggested significantly increased patient satisfaction after implant‐supported retention was provided for the maxillary removable prostheses. With no metal reinforcement, however, the maxillary removable prostheses at patient level had incurred 44% prosthetic complications at 5 years (Bouhy et al. 2023). Overdentures with reduced palatal coverage provide advantages of improved taste perception and retention over conventional dentures. The metal palatal reinforcement in the prostheses likely contributed to the favourable implant and prosthodontic outcomes, particularly important when opposing a natural dentition or implant‐supported prostheses (Sude et al. 2025). Aesthetic, functional and social factors as well as social and socio‐dental dimensions are suggested as important OHQL modulators for acceptance by maxillary IOD users (Lo‐Sardo et al. 2025). Patients were equally satisfied with long‐bar overdentures with and without palate when these are opposed by mandibular fixed prostheses (De Albuquerque Júnior et al. 2000)
Lack of literature	Lack of long‐term RCTs
Evidence	Based on 4 observational studies (De Albuquerque Júnior et al. 2000; Heydecke et al. 2003; Lo‐Sardo et al. 2025; Sude et al. 2025), 1 prospective case series (Bouhy et al. 2023)
Recommended ClinROs	History of patient adherence/compliance Implant primary stability Presence and width of keratinised tissue Surgical/intraoperative complications
Recommended PROs	Aesthetic satisfaction Chewing function/comfort/discomfort Ease of cleaning/oral hygiene efficacy Functional comfort Pain Patient overall satisfaction with treatment Patient‐reported complaints Prosthesis complications Prosthesis retention/stability Speech/phonetics/pronunciation function Quality of life (Oral Health‐Related Quality of Life, OHRQoL)
Strength of Consensus	Agree: 92% (consensus) Agree: 72/Disagree: 4/Abstain: 2/Abstain (CoI): 0

Recommendation No. 9 (Aligned with current evidence)

**Table:** 

Domain	Treatment procedure
Recommendation	In patients with edentulous maxillae, where an implant‐supported fixed dental prosthesis is planned, we recommend a screw‐retained prosthesis for retrievability and designed with contours that enable cleanability. This facilitates maintaining long‐term peri‐implant health
Expert survey results	There was expert consensus (93.4%) for screw‐retained prostheses. Only 4.1% preferred cemented prostheses, and 2.5% selected other options
Patient/cross‐disciplinary expert surveys	Not covered
Supporting/contradicting literature	Screw‐retained reconstructions seem to be preferable to cemented reconstructions. They are more readily retrievable and technical and biological complications can therefore be treated more easily (Sailer et al. 2012). Screw‐retained reconstructions exhibited fewer technical and biologic complications overall (Wittneben et al. 2014). Excess cement has been identified as a potential risk indicator for peri‐implant disease, particularly when soft tissue healing periods are shorter than 4 weeks. As residual cement is more difficult to control in submucosal locations, screw‐retained restorations are preferable whenever feasible, since they eliminate the risk of cement remnants. In situations where cementation cannot be avoided, placing the crown margin at the mucosal level to allow sufficient access, ensuring adequate soft tissue maturation and scheduling early follow‐ups after restoration placement are strongly recommended (Staubli et al. 2017). Screw‐retained restorations (including use of angled abutments and angled screw channels) avoid cement and allows retrievability to address complications (biological, mechanical/technical and loss of proximal contact) (Curtis et al. 2019) If possible, prosthetic reconstructions should be designed and installed with consideration of electing screw‐retained restorations and avoiding local risk factors that may prevent access for oral hygiene (Herrera et al. 2023)
Lack of literature	N/A
Evidence	Based on 3 systematic reviews (Sailer et al. 2012; Wittneben et al. 2014; Staubli et al. 2017) and 2 consensus reports (Herrera et al. 2023; Curtis et al. 2019)
Recommended ClinROs	History of patient adherence/compliance Mechanical/technical complications Presence of keratinised mucosa Prosthodontic maintenance events/complications Width of keratinised mucosa
Recommended PROs	Ease of cleaning/oral hygiene efficacy Patient‐reported complaints Pain
Strength of Consensus	Agree: 95% (consensus) Agree: 74/Disagree: 3/Abstain: 1/Abstain (CoI): 0

Recommendation No. 10 (Aligned with current evidence)

**Table:** 

Domain	Treatment procedure
Recommendation	In patients with edentulous maxillae, receiving fixed implant‐supported prosthesis and subject to evaluation of restorative risk factors, we suggest using a metal framework with traditional ceramic veneering or a milled metal bar with attached milled monolithic/micro‐veneered zirconia as preferred option for a definitive prosthesis. Where appropriate implant distribution is achieved with minimal cantilever, the use of monolithic zirconia is an option. This reduces the risk of technical complications and associated costs
Expert survey results	The expert responses were heterogenous with 39.7% preferring full zirconia; 31.4% zirconia superstructure with a custom titanium framework; 11.6% ceramo‐metal material; 9.9% acrylic over metal framework; 4.1% full zirconia without titanium bases or abutments and 3.3% selecting other
Patient/cross‐disciplinary expert surveys	39% of patients and 42.9% of cross‐disciplinary experts indicated concern regarding risk of prostheses failure
Supporting/contradicting literature	For implant‐ supported fixed dental prostheses, conventionally veneered zirconia should not be considered as material selection of first priority, as pronounced risk for framework fractures and chipping of the zirconia veneering ceramic was observed. Hence, metal ceramics seem to stay the golden standard for implant‐supported multiple‐unit FDPs (Sailer et al. 2018). The predominant technical complication at multiple‐unit, implant‐supported fixed dental prostheses is fracture/chipping of the veneering ceramic (Sailer et al. 2022). Veneering material fractures were the most frequent technical complication (35.8%) (Ng et al. Forthcoming) Subject to further studies with longer follow‐up, the reported high success rates and low incidence of complications in a 2‐year prospective clinical trial involving 193 implant‐supported, full‐arch, fixed prostheses constructed in ceramic‐veneered‐zirconia (83) and monolithic zirconia with buccal surface only veneering (110), suggests zirconia as a suitable material for full‐arch rehabilitations (Caramês et al. 2019)
Lack of literature	Monolithic zirconia may be an interesting alternative, but its clinical medium‐ to long‐ term outcomes have not been evaluated yet (Sailer et al. 2018).
Evidence	Based on 2 systematic reviews (Sailer et al. 2018; Ng et al. Forthcoming) and 1 narrative review (Sailer et al. 2022) and 1 comparative study (Caramês et al. 2019)
Recommended ClinROs	Mechanical/technical complications Prostheses success Prostheses survival Prostheses failure Prosthetic complications Prosthodontic maintenance events/complications
Recommended PROs	Aesthetic satisfaction Chewing function/comfort/discomfort Complications during treatment/maintenance Ease of cleaning/oral hygiene Patient‐reported complaints
Strength of Consensus	Agree: 84% (consensus) Agree: 63/Disagree: 7/Abstain: 5/Abstain (CoI): 0

Recommendation No. 11 (Aligned with current evidence)

**Table:** 

Domain	Treatment procedure
Recommendation	In patients with edentulous maxillae, an accurate final impression/scan can be achieved with conventional analogue or digital scanning techniques. However, we suggest this to be followed with an intraoral verification index. This serves to confirm fit and reduce the risk of biological and technical complications
Expert survey results	The expert responses regarding fabricating the master model were heterogenous with 41.3% preferred splinted open‐tray impression copings, 26.4% intraoral scan, 16.5% non‐splinted open‐tray copings, 8.3% photogrammetry with intraoral scanning, 3.3% closed‐tray impression copings and 4.2% chose other methods
Patient/cross‐disciplinary expert surveys	Most of the patients (58.5%) and cross‐disciplinary experts (52.3%) did not have a preference but 41.5% of patients and 42.9% cross‐disciplinary experts preferred a digital scan
Supporting/contradicting literature	Precision of complete‐arch digital scans, however, depends on several factors of which the following parameters critically influence the reliability of the digital workflow in implant prosthodontics: the distance between implants, the design of the scan bodies, the scanning strategy or path and the operator's clinical experience (Wulfman et al. 2020). Accuracy of intraoral scan is clinically acceptable in edentulous arches, especially for unparalleled implants, but more clinical studies are needed to verify the present finding (Cai et al. 2024)
Lack of literature	Lack of robust evidence
Evidence	Based on 1 systematic review and meta‐analysis (Cai et al. 2024), 1 systematic review (Wulfman et al. 2020)
Recommended ClinROs	Biological complications Mechanical/technical complications Peri‐implant health (implant level) Prosthesis survival Prosthodontic maintenance evens/complications
Recommended PROs	Complications during treatment Patient‐reported complaints
Strength of Consensus	Agree: 93% (consensus) Agree: 71/Disagree: 3/Abstain: 2/Abstain (CoI): 0

Recommendation No. 12 (Could not be evaluated due to a lack of existing evidence)

**Table:** 

Domain	Treatment procedure
Recommendation	In patients with edentulous maxillae, restored with an implant‐supported fixed dental prosthesis, we suggest the delivery of an occlusal guard to reduce the incidence of prosthetic complications, particularly in patients with history of parafunctional habits
Expert survey results	In absence of parafunctions, 39.0% of the experts in the Group 2 survey recommended occlusal guards always and 45.8% in selected cases, mainly based on the status of the opposing dentition or the restoration material
Patient/cross‐disciplinary expert surveys	If offered an occlusal guard to wear at night, 65.9% of patients would wear them daily, 12.2% occasionally and 21.9% rarely. Amongst cross‐disciplinary experts, 66.7% reported daily use, 19.0% occasional use and 14.3% infrequent use
Supporting/contradicting literature	Recommendations exist for occlusal guards for bruxing dentate patients to minimise unfavourable loads and occlusal wear of restorative materials (Berzaghi et al. 2025; Sutthiboonyapan and Wang 2019)
Lack of literature	There is a significant lack of high‐quality evidence to support the routine use in bruxers following dental implant rehabilitation (Häggman‐Henrikson et al. 2024; Mesko et al. 2014; Sutthiboonyapan and Wang 2019)
Evidence	Based on 1 systematic reviews and meta‐analysis (Häggman‐Henrikson et al. 2024), 1 systematic review (Mesko et al. 2014) 2 narrative reviews (Berzaghi et al. 2025, Sutthiboonyapan and Wang 2019)
Recommended ClinROs	Implant failure Mechanical/technical complications Prostheses success Prostheses survival Prostheses failure Prosthetic complications Prosthodontic maintenance events/complications Radiographic marginal bone level Radiographic marginal bone loss
Recommended PROs	Complications during treatment/maintenance Pain Patient‐reported complaints
Strength of Consensus	Agree: 94% (consensus) Agree: 76/Disagree: 3/Abstain: 2/Abstain (CoI): 0

### 6.5 Maintenance

Recommendation No. 13 (Aligned with current evidence)

**Table:** 

Domain	Maintenance
Recommendation	In patients with edentulous maxillae, following the delivery of the implant‐supported/retained dental prostheses, we recommend recording baseline clinical parameters and intraoral radiographs to establish marginal bone levels. This should be followed by regular monitoring of the peri‐implant health status and provision of supportive peri‐implant care (SPIC). The assessment of the integrity of the prosthesis and prosthetic components, occlusion and function is also recommended. We recommend that the frequency of recall should consider the risk profile of the patient to support long‐term prosthesis function, peri‐implant health, patient comfort and quality of life. If there is insufficient access for diagnosis of peri‐implant health status, and for supportive care, we suggest removal and modification of the prosthesis to improve prosthesis design for better access
Expert survey results	When asked on how many times a year a patient with full‐arch implant‐supported fixed prosthesis should receive professional hygiene care, the heterogenous responses from the Group 2 experts were twice a year (44.9%), followed by once (32.2%) and four times a year (11.9%). After the delivery of the final restoration, most of the Group 2 experts indicated that implant patients were generally offered follow‐up at least once a year or more frequently (38.1% and 58.5%, respectively). There was considerable disagreement amongst the Group 4 experts regarding intervals for removal of the definitive prosthesis for evaluation, hygiene or screw replacement but would do so at various annual intervals or when required by signs and symptoms
Patient/cross‐disciplinary expert surveys	There was also considerable disagreement on routine maintenance practices amongst patients where 19.5% preferred visits every 3 months, 39.0% preferred every 6 months and 41.5% preferred annual visits. By contrast 57.1% of the cross‐disciplinary experts favoured check‐ups more frequently every 3 months, 19.0% preferred every 6 months and 23.9% preferred once a year
Supporting/contradicting literature	Guidelines for regular monitoring of the peri‐implant health status and provision of supportive peri‐implant care (SPIC) (Herrera et al. 2023 S3 Guidelines; Berglundh et al. 2018; Jepsen et al. 2015; Lindhe et al. 2008; Renvert et al. 2018; Sanz et al. 2022) Frequency of SPIC should be established on a case‐by‐case basis according to patient‐, implant‐ and restoration‐based risk profiling and needs (e.g., 3‐, 6‐ or 12‐month intervals) (Herrera et al. 2023 S3 Guidelines). When probing is not possible due to the prosthetic emergence profile it affects the diagnostic accuracy of probing depth (Serino et al. 2013). A treatment concept paper introduced a risk assessment tool, the Implant Disease Risk Assessment (IDRA), which estimates the risk for a patient to develop peri‐implantitis (Heitz‐Mayfield et al. 2020)
Lack of literature	No studies were specifically designed to evaluate SPIC frequency in patients with peri‐implant health (Herrera et al. 2023; Stiesch et al. 2023)
Evidence	Based on 6 consensus reports (Herrera et al. 2023 S3 Guidelines; Berglundh et al. 2018; Jepsen et al. 2015; Lindhe et al. 2008; Renvert et al. 2018; Sanz et al. 2022), 1 observational study (Serino et al. 2013) and 1 concept paper (Heitz‐Mayfield et al. 2020)
Recommended ClinROs	Biological complications Clinician's treatment success Implant failure Implant success Implant survival Mechanical/technical complications Peri‐implant health (implant level) Peri‐implant health (patient level) Peri‐implant implantitis Peri‐implant mucositis Peri‐implant suppuration Plaque index/oral hygiene Presence of keratinised mucosa Prostheses success Prostheses survival Prostheses failure Prosthetic complications Prostheses retention/stability Prosthodontic maintenance events/complications Radiographic marginal bone level Radiographic marginal bone loss Width of keratinised mucosa
Recommended PROs	Aesthetic satisfaction Chewing function/comfort/discomfort Complications during treatment/maintenance Ease of cleaning/oral hygiene Pain Patient overall satisfaction with treatment Patient‐reported complaints Prosthesis retention/stability Speech/phonetics/pronunciation function Quality of life (Oral Health‐Related Quality of Life, OHRQoL)
Strength of Consensus	Agree: 76% (consensus) Agree: 48/Disagree: 15/Abstain: 0/Abstain (CoI): 0

Recommendation No. 14 (Could not be evaluated due to a lack of existing evidence)

**Table:** 

Domain	Maintenance
Recommendation	In patients with an edentulous maxilla who are restored with an implant‐supported/retained prosthesis, we suggest patients perform oral hygiene as a minimum twice daily to maintain long‐term peri‐implant health. The oral hygiene aids suggested by the clinician should be individualised according to the patient's dexterity, prosthesis design and peri‐implant tissue conditions. Oral hygiene aids may include toothbrushes (manual or power brushes), interproximal brushes and dental floss. Oral irrigators can be used as an adjunct to other oral hygiene aids. We recommend “oral hygiene methods be demonstrated by the patient to the oral healthcare professional and periodically reinforced”
Expert survey results	They recommended oral hygiene tools for patient use included super floss (72.7%), proxy brushes (72.7%), electric toothbrushes (61.2%), water flossers (58.7%)
Patient/cross‐disciplinary expert surveys	For home care regimen, the patient survey indicated that their preferred choices for cleaning full‐arch prostheses at home toothbrushes (87.8%, consensus), water flossers (70.7%) and interdental brushes (41.5%). Similarly, the cross‐disciplinary expert survey identified toothbrushes (85.7%, consensus), interdental brushes (71.4%) and water flossers (57.1%) as the most preferred tools
Supporting/contradicting literature	Individually tailored oral hygiene instructions with frequency of home care (Carra et al. 2022; Herrera et al. 2023)
Lack of literature	The evidence remains inconclusive regarding the type of toothbrush to use (e.g., powered or manual toothbrush), or the frequency of toothbrushing that is most effective in maintaining peri‐implant health, highlighting the need for individualised recommendations and further research in this area
Evidence	Based on an SP3 level clinical practice guideline (Herrera et al. 2023) and 1 systematic review and meta‐analysis (Carra et al. 2022)
Recommended ClinROs	Clinician's treatment success History of patient adherence/compliance Implant failure Implant success Implant survival Mechanical/technical complications Plaque index/Oral hygiene Peri‐implant health (implant level) Peri‐implant health (patient level) Peri‐implant implantitis Peri‐implant mucositis Peri‐implant suppuration Presence of keratinised mucosa Prostheses success Prostheses survival Prostheses failure Prosthodontic maintenance event/complications Radiographic marginal bone level Radiographic marginal bone loss Width of keratinised mucosa
Recommended PROs	Complications during treatment/maintenance Ease of cleaning/oral hygiene efficacy Pain Patient overall satisfaction with treatment Patient‐reported complaints Quality of life (Oral Health‐Related Quality of Life, OHRQoL)
Strength of Consensus	Agree: 98% (strong consensus) Agree: 59/Disagree: 1/Abstain: 0/Abstain (CoI): 0

## 7 Summary of Areas for Future Research

Future research in full‐arch implant rehabilitation of the edentulous maxilla should address several key domains (Table 1). Current evidence comparing implant‐supported fixed and removable dental prostheses in edentulous patients remains limited, and greater clarity is needed regarding the respective indications for CDs, IODs and IFCDs in the maxilla.

**TABLE 1:** Proposed topics and PICOs for future research.

Domain	Research focus	P (Population)	I (Intervention)	C (Comparator)	O (Outcomes)	S (Study design)
Patient Selection	Prostheses type	Patients with edentulous maxillae	Rehabilitation with IFCDs.	Rehabilitation with IODs.	PROs Aesthetic satisfaction, Chewing function/comfort/ discomfort, Complications during treatment, Ease of cleaning/oral hygiene efficacy Patient overall satisfaction with treatment, Prosthesis retention/stability, Speech/phonetics/pronunciation function ClinROs Biological complications, Clinician's treatment success, Implant survival/success/failure, Presence/Width of keratinised tissue, Prosthetic complications, Peri‐implant health (patient/implant level) Radiographic marginal bone level/loss	Randomised controlled trial (RCT), prospective case series, cohort studies
Treatment Planning	Implant type selection	Patients with edentulous maxillae receiving IOD or IFCD	Bone‐level implants	Tissue level implants	ClinROs Biological complications Implant survival/success/failure, Prosthetic complications, Peri‐implant health (patient/implant level) Radiographic marginal bone level/loss	RCT, prospective case series, cohort studies
Treatment Planning	Most posterior implant site	Patients with edentulous maxillae receiving IOD or IFCD	Most distal implant position first molar	Most distal implant position second molar	ClinROs Biological complications Implant survival/success/failure, Prostheses type (fixed or removable) Prosthetic complications, Peri‐implant health (patient/implant level) Radiographic marginal bone level/loss	RCT, prospective case series, cohort studies
Treatment Planning	Use of angled screw channel systems	Patients with edentulous maxillae receiving IFCDs	Use of angled screw channel systems	Straight and angled abutments with straight channel systems	ClinROs Mechanical/technical complications, Prosthetic complications/failure/ success, Prosthodontic maintenance events/complications	Prospective case series, cohort studies
Treatment Planning	Loading protocol for provisional protheses	Patients with edentulous maxillae receiving IFCDs	Immediate loading protocols for provisional prostheses	Early and conventional loading protocol for provisional prostheses	PROs Patient overall satisfaction with treatment ClinROs Implant primary stability Mechanical/technical complications, Prosthetic complications/failure/ success, Radiographic marginal bone level/loss	RCT, prospective case series, cohort studies
Treatment Planning	Method of manufacture of provisional prostheses	Patients with edentulous maxillae receiving IFCDs	Provisional prosthesis converted from full‐arch denture	Other methods/types of provisional prosthesis	PROs Aesthetic satisfaction, Complications during treatment, Patient overall satisfaction with treatment ClinROs Clinician's treatment success Mechanical/technical complications, Prosthetic complications/failure/ success, Radiographic marginal bone level/loss	RCT, prospective case series, cohort studies
Treatment Planning	One‐piece of segmented prostheses	Patients with edentulous maxillae receiving IFCDs	Implant‐supported one‐piece fixed prostheses	Segmented multi‐unit fixed prostheses	PROs Aesthetic satisfaction, Complications during treatment, Patient overall satisfaction with treatment ClinROs Clinician's treatment success Mechanical/technical complications, Peri‐implant health (patient/implant level) Prosthetic complications/failure/ success, Prosthodontic maintenance events/complications Radiographic marginal bone level/loss	Prospective case series, cohort studies
Treatment Planning	Preferred prosthesis material	Patients with edentulous maxillae receiving IFCDs	Monolithic Zirconia	Metal ceramics	PROs Aesthetic satisfaction, Patient overall satisfaction with treatment ClinROs Clinician's treatment success, Mechanical/technical complications, Peri‐implant health (patient/implant level) Prosthetic complications/failure/ success, Prosthodontic maintenance events/complications Radiographic marginal bone level/loss	RCT, prospective case series, cohort studies
Treatment Planning	Use of titanium framework/abutments/ bases	Patients with edentulous maxillae receiving IFCDs	Milled titanium framework	Monolithic restorations	PROs Patient overall satisfaction with treatment ClinROs Clinician's treatment success Complications during treatment, Mechanical/technical complications, Peri‐implant health (patient/implant level) Prosthetic complications/failure/ success, Prosthodontic maintenance events/complications Radiographic marginal bone level/loss	RCT, prospective case series, cohort studies
Treatment procedure	Maximum number of implant in a full‐arch digital scan	In patients with edentulous maxillae receiving IFCDs	Digital impression	Variable number of implants	PROs Complications during treatment/maintenance Patient overall satisfaction with treatment ClinROs Accuracy Clinician's treatment success, Prosthetic complications/failure/success, Prosthodontic maintenance events/complications	Prospective case series, cohort studies
Maintenance	Prosthesis Retrieval at Different Recall Intervals	Edentulous patients with screw‐retained IFCD	Defined retrieval and maintenance interval	Alternative recall intervals	PROs Patient overall satisfaction with treatment ClinROs Clinician's treatment success, Prosthetic complications/failure/success, Prosthodontic maintenance events/complications Peri‐implant health, Radiographic marginal bone level/loss, Implant failure/success/survival	RCT
PROs	Validated PROMs	Patients with edentulous maxillae receiving IOD or IFCD	Patients receiving implant‐retained/supported maxillary reconstructions	Pre and post rehabilitation IODs vs. IFCDs CD vs. IOD/IFCD	PROMs for Aesthetic satisfaction, Chewing function/comfort/discomfort, Complications during treatment, Ease of cleaning/oral hygiene efficacy, Pain, Patient overall satisfaction with treatment, Prosthesis retention/stability, Speech/phonetics/pronunciation function Quality of life	RCTs Crossover studies Prospective case series

Similarly, there is little evidence on several factors related to implant planning and prosthodontic design for both fixed and removable rehabilitations, including implant selection. Further investigation into these areas is required. With the rapid evolution of digital technologies, the accuracy, applications, benefits and challenges of digital prosthodontic tools and workflows also warrant deeper exploration.

The role of prosthodontic considerations in effective long‐term maintenance remains underexplored. Survey results showed no consensus on routine screw‐retorquing intervals or hygiene protocols, reflecting gaps also identified in recent international clinical practice guidelines (Herrera et al. 2023).

Finally, standardisation and validation of patient‐reported outcome measures (PROMs) are essential to overcome inconsistent reporting when comparing IFCDs and IODs in fully edentulous patients and to generate more meaningful evidence‐based insights.

## Author Contributions

**Charlotte Stilwell:** writing – original draft, writing – review and editing, supervision, conceptualization. **Ronald E. Jung:** writing – review and editing, supervision, conceptualization. **Florian Beuer:** writing – review and editing. **Giulia Brunello:** writing – review and editing. **Jae‐kook Cha:** writing – review and editing. **Jeanette Chua Keng Ling:** writing – review and editing. **Donald A. Curtis:** writing – review and editing. **Karim El‐Kholy:** writing – original draft, writing – review and editing. **Brian Goodacre:** writing – review and editing. **Lisa Heitz‐Mayfield:** writing – review and editing. **Mohit Kheur:** writing – review and editing. **Sunjai Kim:** writing – review and editing. **Hisatomo Kondo:** writing – review and editing. **Purnima Kumar:** writing – review and editing. **Alejandro Lanis:** writing – review and editing. **David Norre:** writing – review and editing. **Bjarni Pjetursson:** writing – review and editing. **Srinivasa Rao Bhasmey:** writing – review and editing. **Irena Sailer:** writing – review and editing. **Misi Si:** writing – review and editing. **Amerian Sones:** writing – review and editing. **Franz J. Strauss:** writing – review and editing. **Stefan Wolfart:** writing – review and editing. **Nicola U. Zitzmann:** writing – review and editing.

## Conflicts of Interest

All delegates disclosed secondary interests using the standardised ICMJE disclosure form. Potential conflicts of interest (CoI) were actively managed in accordance with Guidelines International Network (GIN) principles.

## Data Availability

Data sharing not applicable to this article as no datasets were generated or analysed during the current study.
